# Evaluating Quality, Usability, Evidence-Based Content, and Gamification Features in Mobile Learning Apps Designed to Teach Children Basic Life Support: Systematic Search in App Stores and Content Analysis

**DOI:** 10.2196/25437

**Published:** 2021-07-20

**Authors:** Nino Fijačko, Ruth Masterson Creber, Lucija Gosak, Gregor Štiglic, Dominic Egan, Brian Chaka, Nika Debeljak, Matej Strnad, Pavel Skok

**Affiliations:** 1 Faculty of Health Sciences University of Maribor Maribor Slovenia; 2 Healthcare Policy and Research, Division of Health Informatics Weill Cornell Medicine New York, NY United States; 3 Faculty of Electrical Engineering and Computer Science University of Maribor Maribor Slovenia; 4 Usher Institute University of Edinburgh Edinburgh United Kingdom; 5 School of Nursing and Healthcare Leadership University of Bradford Bradford United Kingdom; 6 School of Allied Health Professions and Midwifery University of Bradford Bradford United Kingdom; 7 Faculty of Medicine University of Maribor Maribor Slovenia

**Keywords:** cardiopulmonary resuscitation, basic life support, mobile learning, mobile phone, gamification, schoolchildren

## Abstract

**Background:**

Globally, 3.7 million people die of sudden cardiac death annually. Following the World Health Organization endorsement of the *Kids Save Lives* statements, initiatives to train school-age children in basic life support (BLS) have been widespread. Mobile phone apps, combined with gamification, represent an opportunity for including mobile learning (m-learning) in teaching schoolchildren BLS as an additional teaching method; however, the quality of these apps is questionable.

**Objective:**

This study aims to systematically evaluate the quality, usability, evidence-based content, and gamification features (GFs) of commercially available m-learning apps for teaching guideline-directed BLS knowledge and skills to school-aged children.

**Methods:**

We searched the Google Play Store and Apple iOS App Store using multiple terms (eg, *cardiopulmonary resuscitation* [*CPR*] or *BLS*). Apps meeting the inclusion criteria were evaluated by 15 emergency health care professionals using the user version of the Mobile Application Rating Scale and System Usability Scale. We modified a *five-finger* mnemonic for teaching schoolchildren BLS and reviewed the apps’ BLS content using standardized criteria based on three CPR guidelines. GFs in the apps were evaluated using a gamification taxonomy.

**Results:**

Of the 1207 potentially relevant apps, only 6 (0.49%) met the inclusion criteria. Most apps were excluded because the content was not related to teaching schoolchildren BLS. The mean total scores for the user version of the Mobile Application Rating Scale and System Usability Scale score were 3.2/5 points (95% CI 3.0-3.4) and 47.1/100 points (95% CI 42.1-52.1), respectively. Half of the apps taught hands-only CPR, whereas the other half also included ventilation. All the apps indicated when to start chest compressions, and only 1 app taught BLS using an automated external defibrillator. Gamification was well integrated into the m-learning apps for teaching schoolchildren BLS, whereas the *personal and fictional*, *educational*, *and performance* gamification groups represented most GFs.

**Conclusions:**

Improving the quality and usability of BLS content in apps and combining them with GFs can offer educators novel m-learning tools to teach schoolchildren BLS skills.

## Introduction

### Background

Sudden cardiac arrest is a leading cause of mortality, responsible for 3.7 million deaths per year [1-5]. Most deaths occur in the community and can be prevented with basic life support (BLS) [6], specifically cardiopulmonary resuscitation (CPR), which doubles the chances of survival [6-8]. The European Resuscitation Council (ERC) [9] and American Heart Association (AHA) [10] guidelines recommend that lay persons respond immediately after a patient collapses and before the arrival of emergency paramedic personnel; however, CPR bystander response rates are <50%, primarily because of low self-efficacy and knowledge of safely conducting CPR by the lay public [6,11,12].

Following the World Health Organization endorsement of the *Kids Save Lives* statement [13], initiatives to include BLS training in primary and secondary schools have been implemented in the hope of increasing rates of bystander CPR [14-16]. Early findings demonstrate that when schoolchildren were educated in BLS, bystander rates of CPR have doubled [17]. Proponents of educating schoolchildren in BLS can do so using interactive digital technologies, including mobile learning (m-learning) [18,19] with gamification features (GFs) [20,21]. Gamification, popularly defined as “the use of game design elements in non-game contexts,” [20] has emerged as a means of harnessing competitiveness by integrating gamification elements such as leaderboards, rewards, badges, avatars, and competitions to engage and motivate consumers [21,22]. The most common tools for teaching BLS to schoolchildren include self-made games [23-25], posters [26], songs [27], and manikins [23,28,29].

### Objectives

The ERC guidelines for resuscitation recommend that the use and development of technology and social media should be encouraged and the impact, assessed [30]. Reviews of apps offering real-time instructions for adult learning and bystander CPR have been published [31,32]; however, they exclude school-aged children. This study aims to systematically evaluate the quality, usability, evidence-based content, and gamification features of commercially available m-learning apps for teaching guideline-directed BLS knowledge and skills to school-aged children.

## Methods

### Searching, Screening, and Reviewing of Commercially Available Apps

We conducted a systematic search of commercially available apps using a rigorous methodology that has been previously published [33,34]. The PRISMA (Preferred Reporting Items for Systematic Reviews and Meta-Analyses) checklist [35] is available in Multimedia Appendix 1. We searched the Google Play Store [36] (for Android apps) and the App Store (for Apple iOS apps) [37] in May 2020. We created inclusion criteria including the *population* (schoolchildren aged 6-13 years), *intervention* (free apps without in-app purchases that contain GFs for schoolchildren to learn CPR), and *outcomes* (app contents for teaching CPR by emergency health care professionals) [38].

**Figure 1:**
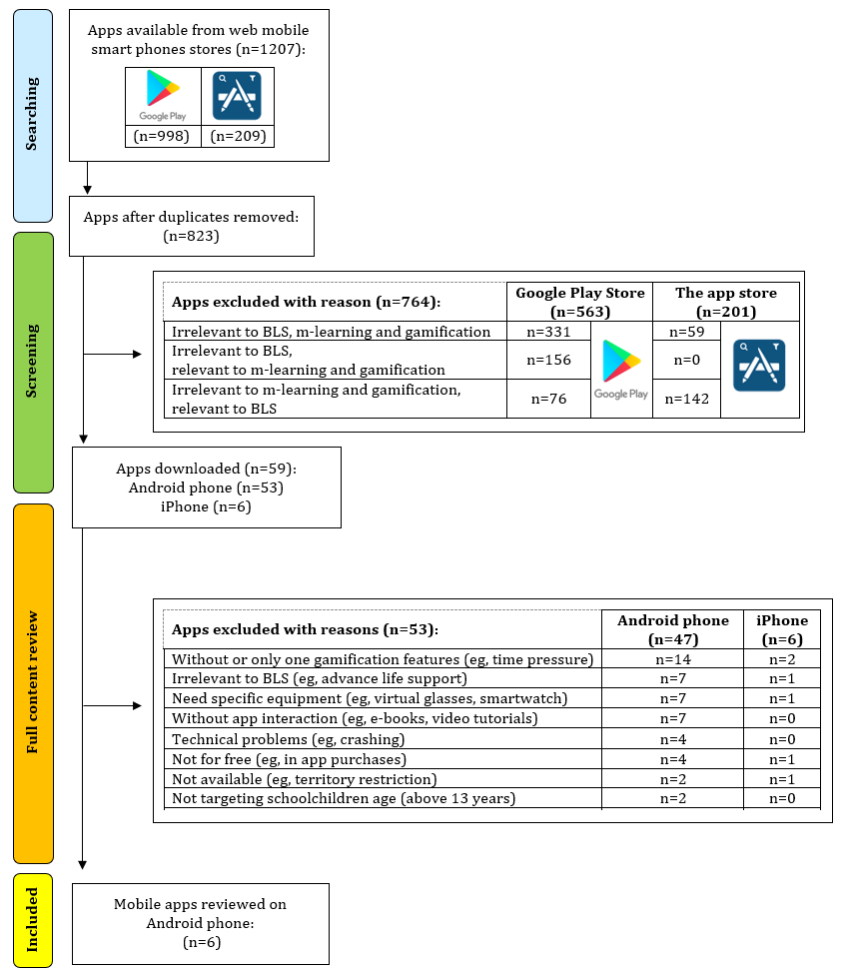
PRISMA (Preferred Reporting Items for Systematic Reviews and Meta-Analyses) flow diagram of app selection. BLS: basic life support.

Our search strategy was conducted in three rounds. First, the apps were searched using search strings (*cardiopulmonary resuscitation*, *CPR BLS*, *CPR BLS kids game*, *CPR BLS game*, and *CPR BLS kids*). After removing duplicates, the apps were screened based on their title, icons, screenshots, photography, pictures, videos, and descriptions by 2 independent investigators. During this round, apps were excluded based on three criteria: (1) irrelevant to BLS, m-learning, and gamification; (2) irrelevant to BLS, relevant to m-learning and gamification; and (3) irrelevant to m-learning and gamification, relevant to BLS. In the third round, the apps were downloaded onto the Samsung Galaxy S8 (Android 9.0 *Pie*) and iPhone 7 (iOS 12.3.1 Apple Inc) mobile phones for Android and iOS apps, respectively, and content was fully reviewed. During this round, apps were excluded based on nine criteria: (1) without or only one GF, (2) irrelevant to BLS, (3) need of specific equipment, (4) without app interaction, (5) technical problems, (6) not for free, (7) not available, (8) not targeting schoolchildren age, and (9) not in English. To ensure consistency, when discrepancies arose, a consensus was reached through discussion by the researchers. The PRISMA flow diagram [35] was used to represent the selection of the included and excluded apps (Figure 1). If the apps were found in both the Google Play Store [36] and Apple App Store [37], they were reviewed in the Google Play Store [39].

### Evaluator’s Recruitment

The inclusion criteria for the selection of emergency health care professionals were as follows: above 18 years, CPR training certification by an established medical association, and more than 5 years of experience teaching emergency medicine. A total of 15 emergency health care professionals rated each app independently in a laboratory environment using two validated rating tools, the user version of the Mobile Application Rating Scale (uMARS) [40] and System Usability Scale (SUS) [41]. The duration of each app review was recorded, and 3 additional investigators with expertise in consumer health informatics and emergency medicine reviewed each app and rated them for BLS content.

### Ethics Approval

The study was conducted in a central European country (Slovenia). All emergency health care professionals signed informed consent to participate, and ethics approval was obtained from the two Health Care Centre in the north-eastern part of Slovenia.

### Measures and Rating Tools

#### Rating Tools for App Quality and Usability

uMARS provides a multidimensional measure of performance indicators, functionality, esthetics, and quality of information. Apps’ subjective quality was not assessed. All items were rated on a 5-point Likert scale ranging from 1 (inadequate) to 5 (excellent). The SUS includes 10 statements on a 5-point Likert scale with both positive and negative statements about usability. The total score for the SUS is 100 points, which is divided into six usability categories including worst imaginable (0-25 SUS score), poor (25.1-51.6 SUS score), ok (51.7-62.6 SUS score), good (62.7-72.5 SUS score), excellent (72.6-84.0 SUS score), and best imaginable (84.1-100 SUS score) [42-44]. The uMARS and SUS have been used in similar studies [31,32,34,45-47].

#### Rating Tool for BLS-Related Content Based on CPR Evidence

In total, 3 investigators evaluated evidence-based BLS in each app using the ERC [9,48], AHA [10,49], and Australian Resuscitation Council [50] guidelines for teaching BLS. On the basis of these guidelines, the team identified 17 discrete BLS contents and divided them into five groups based on the Slovenian Resuscitation Council *five-finger* BLS teaching mnemonic for teaching first responders BLS [51]: (1) *safety* (1 item, BLS1), (2) *consciousness* (2 items, BLS2-3), (3) *breathing and call* (4 items, BLS4-7), (4) *CPR* (9 items, BLS8-16), and (5) *defibrillation* (1 item, BLS17), as shown in Multimedia Appendix 2 [9,10,48,49,51]. The *five-finger* BLS teaching mnemonic is based on two well-known ways of remembering CPR: (1) *DRSABCD* (pronounced *drs A-B-C-D*; danger, responses, send, airway, breathing, CPR, defibrillation) [52] action plan and (2) *chain of survival* [53]. The scoring system included one point if the BLS content was correctly implemented based on the BLS guidelines. We used a digital metronome to compare the frequency of chest compressions in apps.

#### Rating Tool for GFs in Apps

We modified gamification taxonomy [54,55] into five gamification groups, where each group represented different GFs: (1) *ecological* (4 GFs, GF1-4), (2) *social* (3 GFs, GF5-7), (3) *personal and fictional* (7 GFs, GF8-14), (4) *performance* (8 GFs, GF15-22), and (5) *educational* (4 GFs, GF23-26). The purpose of this gamification taxonomy is to evaluate m-learning environments such as apps. Each gamification group has a different relationship with the environment and learners in the form of implementation (ecological group), interaction (social group), usage (personal and fictional group), response (performance group), and knowledge (educational group). A total of 26 GFs were included in the final gamification taxonomy, as presented in Multimedia Appendix 3 [54,55]. The gamification rating was classified using a dummy coding [56] by 2 investigators, one point for inclusion of gamification taxonomy, no points for no gamification taxonomy, and 0.5 points for partial implementation of gamification taxonomy.

### Data Analysis

We used Microsoft Office Professional 2016, R (version 3.6.0, R Foundation for Statistical Computing), SPSS Statistics for Windows (version 27.0, IBM Corp), and Inkscape 1.0 (Inkscape Developers, GNU General Public License) to analyze and visualize the results. The interrater reliability for uMARS and SUS was calculated using the intraclass correlation coefficient (ICC_2, k_; intraclass correlation coefficient, two-way random, average measures, and absolute agreement) [57,58].

## Results

### Overview

We identified 1207 apps. The PRISMA flow diagram presents the process of selecting and scanning apps using the inclusion and exclusion criteria. After removing duplicates from multiple search strings from web-based mobile smartphone stores, 63.29% (764/1207) apps remained; 4.88% (59/1207) apps were relevant to BLS, m-learning, and gamification. After applying all the inclusion and exclusion criteria, 0.49% (6/1207) apps were included in the final evaluation (Figure 1).

All apps were classified into the *educational* category by the Google Play Store. Half of the apps had a disclaimer that the app was made for educational purposes only and was not a substitute for accredited BLS training. Only 2 apps required registration. Apps were developed across multiple countries, including Australia, Italy, Finland, China, and the United States. All apps were developed in collaboration with one or more health care organizations (Table 1 and Multimedia Appendix 4).

**Table 1:** Description of the included apps.

Full App Name	Health care organization collaborator	Country, BLS^a^ guideline organization	Description of BLS scenarios
*First Aid Action Hero* [59]	St John Ambulance Australia (Victoria)	Australia (St John Ambulance Australia)	One scenario in which the user performed BLS, first on a conscious and second on an unconscious animated cartoon figure
*CPR APP* [60]	Emergency Medicine Unit, Li Ka Shing Faculty of Medicine, The University of Hong Kong	United States (American Heart Association)	No scenario. In the simulation environment, the user performed BLS on an animated human figure
*Everyday Lifesaver* [61]	Life Saving Victoria Limited	Australia (St John Ambulance Australia)	Three scenarios in which the user performed BLS on a drowned adult, a drowned child, and an unconscious animated cartoon figure
*A Breathtaking Picnic* [62]	The Italian Resuscitation Council	Italy (The Italian Resuscitation Council)	Two scenarios in which the user performed BLS on an animated animal that was choking and one that experienced cardiac arrest
*ReLIVe Responder* [63]	The University of Pittsburgh, Department of Emergency Medicine	United States (American Heart Association)	Two scenarios in which the user performed BLS on an unconscious and a conscious animated human figure
*Responder Rescuebusters: Fire and First-Aid* [64]	Emergency Response Centre Agency Finland, Finnish Recovery Council, Finnish Fire Officers’ Association’s	Finland (Finnish Recovery Council)	One scenario in which the user performed BLS on an animated human figure that experienced cardiac arrest

^a^BLS: basic life support.

Most apps targeted children aged above 4 years of age, and 1 app—*A Breathtaking Picnic* [62]—targeted schoolchildren aged between 6 and 8 years. All the apps had a Pan European Game Information 3 certificate [65]. According to game genres [65], 3 apps were developed as animated tutorials, 2 apps were developed as simulations, and 1 app was developed as a virtual world; 2 apps represented the first responder as virtual characters (eg, animated boy or animal), and an animated victim was included in each app (Table 1).

### uMARS Quality and SUS Usability Rating

A total of 15 emergency health care professionals participated (3 females and 12 males) in evaluating apps using uMARS and SUS. In total, 40% (6/15) of the participants were nurses, 27% (4/15) were nurses with a master’s degree, and 33% (5/15) were physicians. Overall, the mean age of emergency health care professionals was 36 years. All emergency health care professionals had an Advanced Life Support (ALS) certificate provided by the ERC, and their mean professional experience was 13 years. All emergency health care professionals own and were proficient daily users of mobile smartphones.

**Figure 2:**
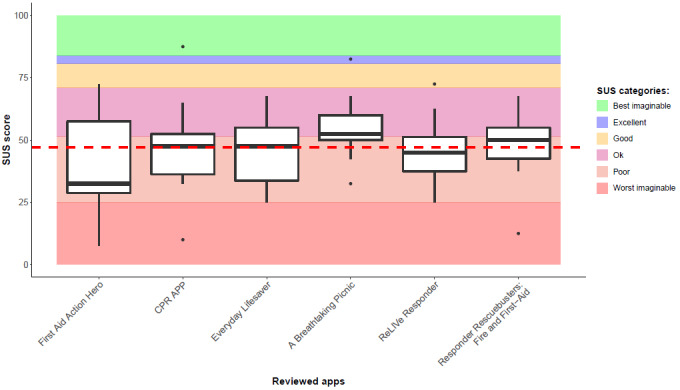
System Usability Scale results in the form of a box plot for each review app. SUS: System Usability Scale.

The mean total uMARS rating of apps was 3.2/5 (95% CI 3.0-3.4), and the details across the four domains are shown in Multimedia Appendix 5. The mean testing app time was 9.5 minutes. The most time-consuming app was the *Everyday Lifesaver* [61] app (mean 24 minutes) because the app included multiple features for evaluation. Interrater reliability between emergency health care professionals was good for the overall uMARS score (ICC_2,k_ 0.8, 95% CI 0.8-0.9; Tables 2 and 3; Multimedia Appendix 6) but poor for overall SUS score (ICC_2,k_ 0.3, 95% CI 0.03-0.5). The *A Breathtaking Picnic* [62] app had the highest mean SUS score (54.8 points). The mean SUS score of all assessed apps was 47.1/100 (95% CI 42.1-52.1) points. The usability of the apps was rated from *poor* to *ok*. The mean SUS score of the apps is indicated by a red dashed line. The six bands in the Figure 2 indicate the six levels of SUS categories of usability.

**Table 2:** User version of the Mobile Application Rating Scale results and time spent on each app.

Full app name	uMARS^a^ section						Time^b^
	Engagement	Functionality	Aesthetics	Information	Overall app quality		
*First Aid Action Hero* [59]	3.7	4.0	4.0	3.9	3.9	12	
*CPR APP* [60]	3.1	3.7	3.2	3.7	3.4	7	
*Everyday Lifesaver* [61]	3.5	3.0	3.7	3.5	3.4	24	
*A Breathtaking Picnic* [62]	2.9	3.5	4.1	3.7	3.5	5	
*ReLIVe Responder* [63]	2.8	3.6	3.2	3.5	3.3	5	
*Responder Rescuebusters: Fire and First-Aid* [64]	3.4	3.4	3.6	3.2	3.4	4	

^a^uMARS: user version of the Mobile Application Rating Scale.

^b^Mean time for testing apps (in minutes).

**Table 3:** Mean scores and intraclass correlation coefficients of the user version of the Mobile Application Rating Scale and time spent on each app.

Variable			Score, mean (95% CI)		ICC_2, k_^a^ (95% CI)
**uMARS^b^ section**					
	Engagement	3.2 (3.0-3.4)		0.9 (0.8-0.9)	
	Functionality	3.5 (3.4-3.7)		0.7 (0.5-0.8)	
	Aesthetics	3.6 (3.4-3.8)		0.8 (0.6-0.9)	
	Information	3.6 (3.4-3.8)		0.8 (0.6-0.9)	
	Overall app quality	3.2 (3.0-3.4)		0.9 (0.8-0.9)	
Time for testing apps (min)			9.2 (7.7-10.7)		N/A^c^

^a^ICC_2,k_: intraclass correlation coefficient; two-way random, average measures, absolute agreement.

^b^uMARS: user version of the Mobile Application Rating Scale.

^c^N/A: not applicable.

### Evidence-Based BLS Content

**Figure 3:**
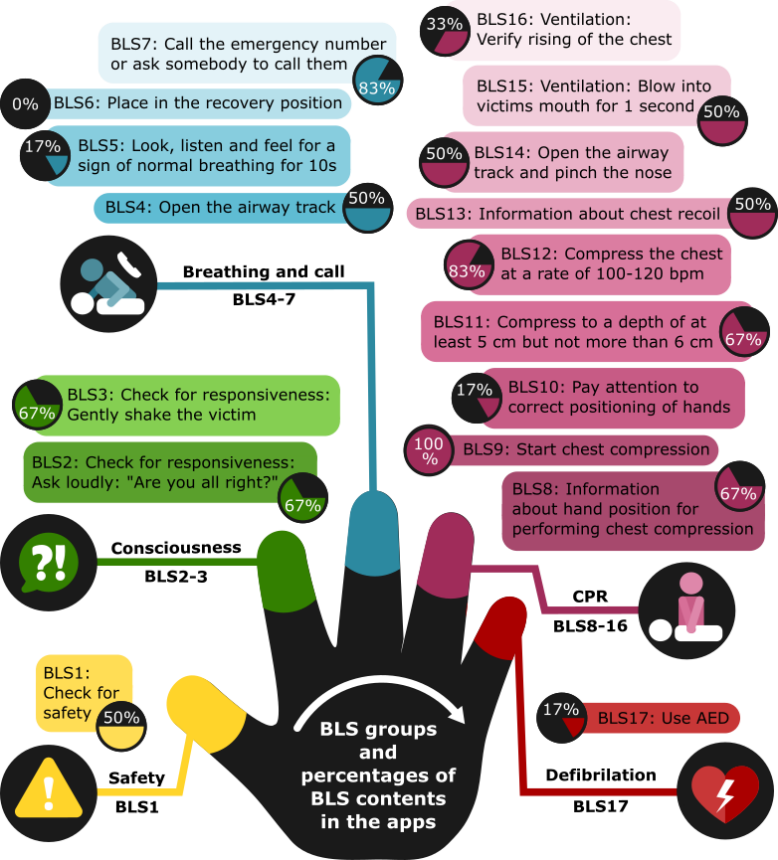
Basic life support groups and percentages of basic life support contents in the apps. AED: automated external defibrillator; BLS: basic life support; CPR: cardiopulmonary resuscitation.

The overall evaluation of evidence-based BLS content in the apps was *poor* to *average* based on expert consensus. Within each of the five categories, there were inconsistencies regarding mapping to the ERC [9,48], AHA [10,49], and Australian Resuscitation Council [50] guidelines for teaching BLS (Figure 3 and Multimedia Appendix 2). In the *safety* category, 50% (3/6) of the apps included checking for safety. For *consciousness*, most apps (4/6, 67%) included checking for responsiveness. Within the *breathing and call* category, the vast majority (5/6, 83%) included calling the emergency number or asking someone to call them; however, none of the apps included placing the patient in the right recovery position. In the *CPR* category, half of the apps taught hands-only CPR, whereas the other half also included ventilation, which is inconsistent with the most recent BLS guidelines internationally. Only 1 app included teaching BLS using an automated external defibrillator (AED). In the 4 apps, the chest compression frequency was set to 100 beats per minute.

### Gamification Features

**Figure 4:**
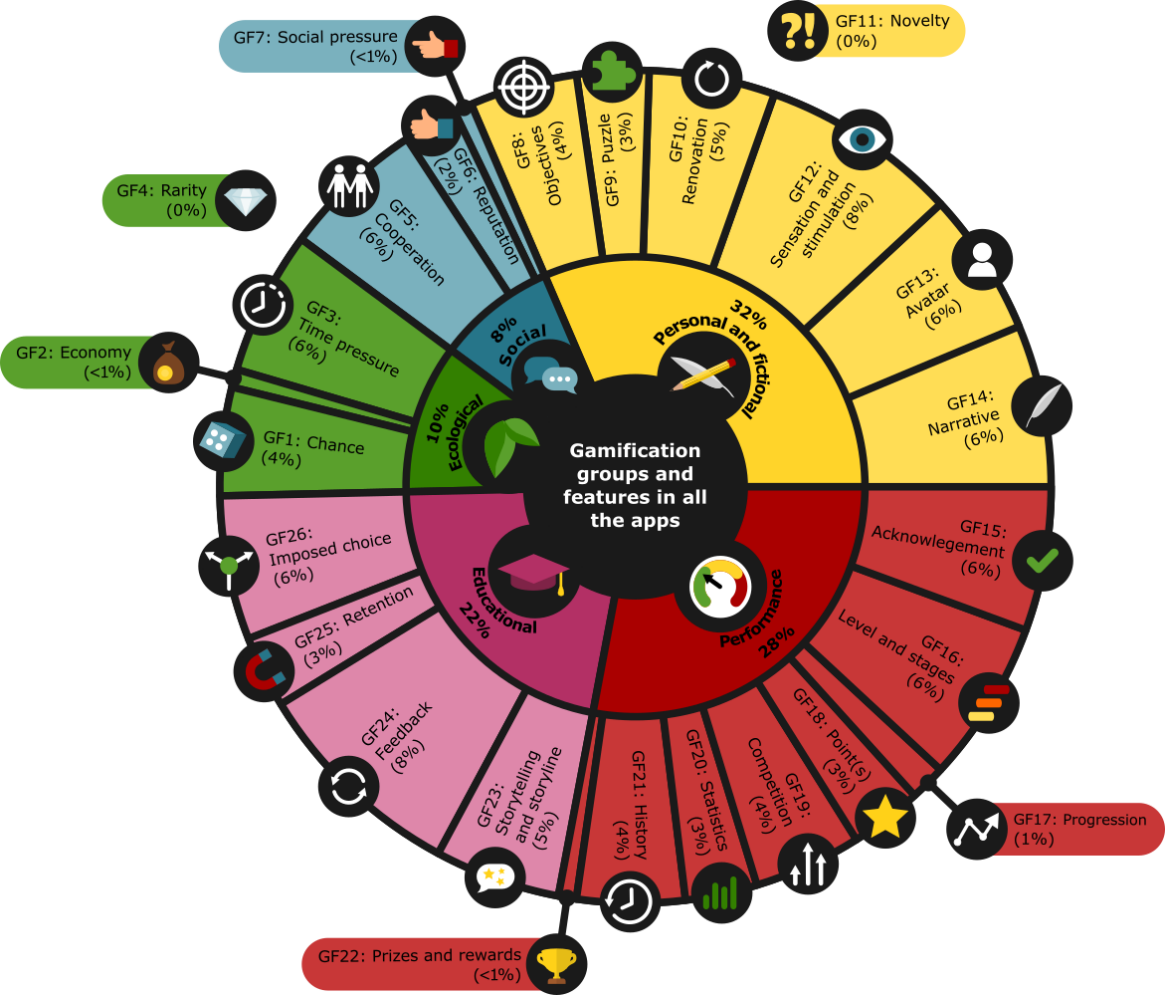
Gamification groups and features in all the apps. GF: gamification feature.

The most common GFs in apps were in *personal and fictional* (32%), *performance* (28%), and *educational* (22%; Figure 4 and Multimedia Appendix 3) gamification groups. One of the most integrated GFs in the apps for teaching schoolchildren BLS was feedback and sensation or stimulation (both 8%).

## Discussion

### Principal Findings

We conducted a systematic evaluation of the quality, usability, evidence-based content, and GFs of commercially available m-learning apps for teaching guideline-directed BLS knowledge and skills to school-aged children. Overall, the quality of the apps was *average* based on the uMARS, the usability was *poor* to *ok* based on the SUS, and the quality of the content was *poor* to *average* in terms of alignment with international BLS guidelines, and GFs were well represented across the gamification taxonomy.

### Quality and Usability of Apps

Many of the apps analyzed in this review were not high-quality apps according to the uMARS tool. Overall, the lowest mean uMARS score was represented in the engagement section, evaluating entertainment, interest, customization, interactivity, and target group. Future apps could learn from this review by ensuring that user engagement is prioritized during the development phase. From a customization perspective, some personal app options (eg, selecting gender or adding names of a player) or BLS options such as changing BLS victims or scenarios, including or excluding ventilation as a part of CPR, and varying chest compression frequency should be added. Nevertheless, the information section represented the highest uMARS score, and app developers should consider adding more relevant evidence-based BLS contents to BLS m-learning apps.

Relevant to functionality and esthetics, schoolchildren prefer visually attractive apps [22] with high levels of interactivity [66-68] and a relatable storyline (eg, bicycle accident) that evokes empathy for the victim [26]. However, in this study, the quality of visual esthetics was inversely proportional to the app’s learnability and usability. Of the 6 apps examined with the SUS tool, the app *A Breathtaking Picnic* [62] had the highest mean usability score. Educators must consider a few critical aspects of choosing the most usable m-learning app for teaching BLS to schoolchildren. Currently, there is no single app available that is appropriate for teaching BLS to all children aged 8-18 years. On the basis of this review, we recommend using the *A Breathtaking Picnic* [62] app (54.8 points) for teaching schoolchildren aged 6-8 years, the *First Aid Action Hero* [59] app (40.1 points) for children aged 8-10 years, and the *Everyday Lifesaver* [61] app (45.7 points) for children aged 11-18 years. Apps with SUS scores higher than 82 have a considerable chance of being recommended to a colleague [32]; in this review, none of the apps achieved this score.

### Evaluation of Evidence-Based BLS Content

This review represented the poor-to-average quality of BLS content regarding international BLS guidelines [9,10,48-50].

#### Five-Finger BLS Teaching Mnemonic

We classified BLS training for schoolchildren using a modified version of the *five-finger* BLS teaching mnemonic [26,49], including (1) *safety,* (2) *consciousness,* (3) *breathing and call,* (4) *CPR,* and (5) *defibrillation* (Figure 3).

#### Safety

In terms of safety, only half of the apps were designed to check whether the area was safe before approaching the victim. All BLS guidelines reinforce the importance of ensuring safety for first responders, victims, and bystanders [9,10,48-50].

#### Consciousness

Consciousness, in most apps, was assessed by checking the victim’s responsiveness to the question, “Are you all right?” and gently shaking the victim. For example, in the *Everyday Lifesaver* [61] app, responsiveness is taught using the acronym COWS [50,69] (“Can you hear me?”, “Open your eyes,” “What’s your name?”, and “Squeeze my hand”). Studies have shown that schoolchildren do not have problems in correctly assessing consciousness [24,26,70,71].

#### Breathing and Call

As recommended in the CPR guidelines [9,10,48-50], for the breathing and call category, the head tilt–chin lift maneuver is generally well taught in the apps, except when the jaw has to be lifted upward to bring the chin forward and the teeth almost to occlusion. Most apps included *calling the emergency number or asking somebody to call them*; however, none of the apps correctly showed the process of moving the victim into the right recovery position or turning away from the rescuer (Figure 3). A problem with most apps is teaching the *look, listen*, and *feel* method for signs of breathing discretely. Importantly, in the context of the COVID-19 pandemic, the head tilt–chin lift maneuver and *look*, *listen*, and *feel* method is no longer recommended in the 2020 guidelines [72]. Most apps do not emphasize abnormal types of breath; only the *ReLIVe Responder* [63] app and *Everyday Lifesaver* [61] app provide information about agonal breathing or gasping or gurgling, as was shown in a study conducted in 2018 [32].

Most apps correctly demonstrated how to make an emergency service call using the speakerphone function. For example, in the *Everyday Lifesaver* [61] app game scenario, the mock operator’s questions are based on the 5 Ps (*place, phone number, problem, people, and progress*). Most schoolchildren can correctly remember the information needed to make emergency calls [73-75].

#### CPR

##### Chest Compression

The most common BLS content included in apps was chest compressions. According to the modified CPR guidelines [72], hands-only CPR is recommended to decrease the risk of COVID-19 infection. Chest compressions were typically indicated by a circle on the chest for each compression. The major problem is that the area for pressing frequently does not correspond with the correct anatomical location, and this could provide users with misleading visual information, especially schoolchildren. As such, the biggest limitation of the apps overall is the gestural design of chest compressions, especially because there is no universal gestural design. In addition, GFs focus on users’ attention to pressing a specific circle rather than focusing on the victim. Similarly, in a real scenario, there are no clear indicators of where to compress, and not knowing appropriate anatomical landmarks could create confusion. This is further complicated by the fact that most of the victims were cartoon animals, so specific locations for chest compressions are unclear when translated to humans.

An alternative approach to indicating the point for compression using a circle is to interact with the phone by holding the smartphone in the palm, facing up, and moving it up and down with the chest compressions. This method facilitates immediate visual feedback on how chest compressions should be performed. A limitation of the apps was that the compression site was inconsistently labeled, leading to inaccurate hand and arm positions. According to a study conducted in 2019, consistency with hand and arm positions is critical for chest compression accuracy [76]. According to the CPR guidelines [9,10,48-50], chest compressions should be at least 5-6 cm in depth, at a rate of 100-120 compressions per minute (2 per second). In general, the apps provided appropriate BLS information regarding the depth and rate of chest compression. On average, the frequency of chest compressions was set to 100 beats per minute and could not be changed to higher frequency rates. Only a few apps emphasize chest recoil and fraction. One of the challenges for schoolchildren is having the strength to perform chest compressions [77]. Even if a child is not physically able to perform chest compressions, they can still learn the fundamentals of BLS and are capable of learning comprehensive BLS content and selecting skills [29,78].

##### Ventilation

Our results indicate that half of the apps do not include steps for ventilation when teaching CPR. Those that do include ventilation provide accurate BLS contents about how to perform mouth-to-mouth ventilation. However, ventilation volume and verification of chest rising are poorly integrated into the ventilation part of CPR. A study from 2019 indicated that teaching schoolchildren ventilation requires more teaching time, and it is harder to establish good quality BLS results [76]. Apps that teach ventilation as a part of CPR are more time-consuming; however, they also adhere more closely to the BLS guidelines. In addition, according to the modified CPR guidelines, mouth-to-mouth ventilation is not recommended to decrease the risk of COVID-19 infection [72].

#### Defibrillation

Overall, the use of AEDs was poorly represented. Only the *Everyday Lifesaver* [61] app included AEDs for teaching schoolchildren BLS training. A study reported that using an app that provides AEDs may be beneficial in terms of performance and security but at the cost of delivering a shock [79]. However, a small proportion of the schoolchildren without previous training could use an AED correctly in less than 3 minutes following the device’s acoustic and visual instructions [80].

### GFs in Apps

Recently, gamified m-learning has become increasingly popular in various medical and educational contexts, including BLS training [81-83]. Through gamification, not only can apps create a mindset that encourages schoolchildren to try new things without being afraid of failing [84,85] but it also enables schoolchildren to engage in the learning experience.

*Personal and fictional*, *educational*, and *performance* GFs were the most represented in apps for teaching BLS to schoolchildren. Gamification audio or visual BLS feedback features, levels and stages in the way of BLS steps, and sensation in a sort of stimulation were well integrated into apps for teaching schoolchildren BLS. Knowledge retention plays an important role in teaching BLS [22,86-88]; only two apps (eg, the Everyday Lifesaver [61] app) included knowledge retention in the form of repeated BLS content. It is recommended that retention content be integrated into apps because of the rapid deterioration of BLS skills after training.

Schoolchildren today have high smartphone literacy, but less is known about educators. To use m-learning to teach BLS to schoolchildren, educators must feel confident about the platform. Some resuscitation councils, such as the Italian Resuscitation Council [89] or recently ERC [90], have already recognized that m-learning is a new trend in education and are starting to emphasize m-learning in the future resuscitation teaching guidelines. The most recent ERC draft guidelines [91] recommend that schoolchildren need supervision for learning BLS and that schoolteachers are more appropriately positioned to teach schoolchildren than health care professionals [92,93].

Using gamified learning features, educators can expect changes in psychomotor, cognitive, and affective learning outcomes [82,83,94]. A systematic review [65] demonstrated that knowledge acquisition and retention of content, productive learning experience, and motor skills are all improved when GFs are incorporated into m-learning. Therefore, learning BLS should include both knowledge transfer and the motivation to perform BLS. We conclude that m-learning has the potential to be used to enhance BLS education for schoolchildren by improving the retention of BLS knowledge.

### Limitations

We deliberately selected emergency health care professionals and not schoolteachers to review the apps because we were focused on adherence to evidence-based guidelines for educational purposes. There were also a small number of final apps, probably as a result of our prespecified criteria, including focusing on a target population below 13 years of age and free apps. In addition, a few apps were excluded because of limitations in language design (eg, Held: Reanimatie Game, in Dutch) [95] and the area where the study was conducted (eg, the First Aid Skills app is available only in Australia) [96]. Finally, all of the apps were developed before the COVID-19 pandemic; therefore, COVID-19–specific modifications to BLS content were not included.

### Future Research

The results of this study provide opportunities for developing an app for teaching BLS to schoolchildren. The *First Aid Action Hero* [59] app and *A Breathtaking Picnic* [62] app have potential to be part of a randomized controlled study in which the effects of m-learning on knowledge retention, motor skills, and motivation to perform BLS can be evaluated.

### Conclusions

Our study represents an opportunity to include m-learning apps for teaching BLS to schoolchildren. Using an adapted *five-finger* BLS teaching mnemonic and m-learning with GFs, there is tremendous potential for teaching BLS to schoolchildren to improve survival rates of cardiac arrest.

## Appendix

Multimedia Appendix 1 PRISMA (Preferred Reporting Items for Systematic Reviews and Meta-Analyses) checklist.

Multimedia Appendix 2 Basic life support groups and contents.

Multimedia Appendix 3 Gamification groups and features.

Multimedia Appendix 4 Description of the included apps.

Multimedia Appendix 5 User version of the Mobile Application Rating Scale scores and sections.

Multimedia Appendix 6 User version of the Mobile Application Rating Scale and time spent on each app results.

## Abbreviations

AED: automated external defibrillator
AHA: American Heart Association
ALS: Advanced Life Support
BLS: basic life support
CPR: cardiopulmonary resuscitation
ERC: European Resuscitation Council
GF: gamification feature
ICC: intraclass correlation coefficient
PRISMA: Preferred Reporting Items for Systematic Reviews and Meta-Analyses
SUS: system usability scale
uMARS: user version of the Mobile Application Rating Scale
