# Supplementary material for: Evaluating Quality, Usability, Evidence-Based Content, and Gamification Features in Mobile Learning Apps Designed to Teach Children Basic Life Support: Systematic Search in App Stores and Content Analysis
Source: JMIR Mhealth Uhealth. 2021 Jul 20;9(7):e25437. doi: 10.2196/25437 (PMC8335615; doi:10.2196/25437)
Supplement: Multimedia Appendix 2 [file mhealth_v9i7e25437_app2.docx]

**Multimedia Appendix 2:** Basic life support groups and basic life support contents.

| **BLS^a^ groups** | **BLS contents [9,10,48,49,51]** | | **Apps that included that BLS content** |
| --- | --- | --- | --- |
|  | **ID^b^** | **Name** |  |
| **Safety** | **BLS1** | Check for safety | - First Aid Action Hero - A Breathtaking Picnic - Everyday Lifesaver |
| **Consciousness** | **BLS2** | Check for responsiveness: Ask loudly: “Are you all right?“ | - First Aid Action Hero - A Breathtaking Picnic - Responder Rescuebusters: Fire and First-Aid - Everyday Lifesaver |
|  | **BLS3** | Check for responsiveness: Gently shake the victim | - First Aid Action Hero - A Breathtaking Picnic - ReLIVe Responder - Responder Rescuebusters: Fire and First-Aid |
| **Breathing and call** | **BLS4** | Open the airway track | - A Breathtaking Picnic - Responder Rescuebusters: Fire and First-Aid - Everyday Lifesaver |
|  | **BLS5** | Look, listen, feel for a sign of normal breathing for 10 s | - Everyday Lifesaver |
|  | **BLS6** | Place in the recovery position | N/A^c^ |
|  | **BLS7** | Call the emergency number or ask somebody to call them | - First Aid Action Hero - A Breathtaking Picnic - ReLIVe Responder - Responder Rescuebusters: Fire and First-Aid - Everyday Lifesaver |
| **CPR^d^** | **BLS8** | Information about hand position for performing chest compression | - First Aid Action Hero - A Breathtaking Picnic - ReLIVe Responder - Everyday Lifesaver |
|  | **BLS9** | Start chest compression | - First Aid Action Hero - CPR APP - A Breathtaking Picnic - ReLIVe Responder - Responder Rescuebusters: Fire and First-Aid - Everyday Lifesaver |
|  | **BLS10** | Pay attention to correct positioning of hands | - CPR APP |
|  | **BLS11** | Compress to a depth of at least 5 cm but not more than 6 cm | - CPR APP - A Breathtaking Picnic - ReLIVe Responder - Everyday Lifesaver |
|  | **BLS12** | Compress the chest at a rate of 100-120 bpm | - First Aid Action Hero - CPR APP - A Breathtaking Picnic - ReLIVe Responder - Everyday Lifesaver |
|  | **BLS13** | Information about chest recoil | - CPR APP - A Breathtaking Picnic - ReLIVe Responder |
|  | **BLS14** | Open the airway track and pinch the nose | - First Aid Action Hero - Responder Rescuebusters: Fire and First-Aid - Everyday Lifesaver |
|  | **BLS15** | Ventilation: Blow into victim’s mouth for 1 second | - First Aid Action Hero - Responder Rescuebusters: Fire and First-Aid - Everyday Lifesaver |
|  | **BLS16** | Ventilation: Verify rising of the chest | - Responder Rescuebusters: Fire and First-Aid - Everyday Lifesaver |
| **Defibrillation** | **BLS17** | Use AED^e^ | - Everyday Lifesaver |

^a^BLS: basic life support.

^b^ID: Identification number

^c^N/A: not applicable (First aid action hero app and Everyday lifesaver app turn the victim on the opposing (wrong) side.).

^d^CPR: cardiopulmonary resuscitation

^e^AED: automated external defibrillator
