# Supplementary material for: Evaluating Quality, Usability, Evidence-Based Content, and Gamification Features in Mobile Learning Apps Designed to Teach Children Basic Life Support: Systematic Search in App Stores and Content Analysis
Source: JMIR Mhealth Uhealth. 2021 Jul 20;9(7):e25437. doi: 10.2196/25437 (PMC8335615; doi:10.2196/25437)
Supplement: Multimedia Appendix 3 [file mhealth_v9i7e25437_app3.docx]

**Multimedia Appendix 3:** Gamification groups and features.

| **Gamification groups** | **Gamification features [54,55]** | | | **Apps with gamification feature** |
| --- | --- | --- | --- | --- |
|  | **ID^a^** | **Name** | **Description** |  |
| Ecological | **GF1** | Chance | Also known as randomness, luck, fortune or probability. This intrinsic concept is related to the random property of a certain event or outcome, eg, the student may get a random number of points after completing a task; spinning a roulette that may give the user a bonus. | - First Aid Action Hero^1^ - A Breathtaking Picnic^1^ - Responder Rescuebusters: Fire and First-Aid - Everyday Lifesaver |
|  | **GF2** | Economy | Also known as transactions, market, exchange. This concept is extrinsically related to any transaction that may occur in the environment. Examples are trading points for advantages within the environment and related to the content. | - Responder Rescuebusters: Fire and First-Aid^1^ |
|  | **GF3** | Rarity | Also known as limited items, collection, exclusivity. It is related to extrinsically limited resources within the environment which can stimulate the learners through a specific goal. | / |
|  | **GF4** | Time Pressure | Also represented as countdown timers or clocks. It is related to time itself used to pressure the learners’ actions (extrinsic). In learning environments, this can be represented also as deadlines. | - First Aid Action Hero - A Breathtaking Picnic - ReLIVe Responder - Responder Rescuebusters: Ffire and First-Aid - Everyday Lifesaver ^1^ |
| Social | **GF5** | Cooperation | Also known as teamwork, co-op, groups, etc. It is also an intrinsic concept (related to a task) where the users must collaborate to achieve a common goal, can be considered the opposite of competition (however, both concepts can be used together). Examples of cooperation are tasks where groups interact with each other and are recognised by these interactions. | - First Aid Action Hero - A Breathtaking Picnic^1^ - ReLIVe Responder - Responder Rescuebusters: Fire and First-Aid^1^ - Everyday Lifesaver |
|  | **GF6** | Reputation | Also known as classification, status. It is related to titles that the learner may gain and accumulate within the environment (intrinsic). Differing from levels, titles represent more of a social status which does not necessarily reflect on the learners’ skills. These titles are usually used within communities to create a hierarchy in the environment. | - First Aid Action Hero^1^ - Responder Rescuebusters: Fire and First-Aid^1^ - Everyday Lifesaver^1^ |
|  | **GF7** | Social Pressure | Also known as peer pressure or guild missions. This intrinsic concept is related to social interactions that exert pressure on the learner. | - ReLIVe Responder^1^ |
| Personal/  Fictional | **GF8** | Novelty | Also known as an update, surprise, changes, etc. It is intrinsically related to the updates that occur within the environment, by adding new information, content or even new game elements. It is a good strategy to keep users within the environment to avoid stagnation since longitudinal studies on gamification have shown that a static approach (without updates) may cause disengagement and demotivation. | / |
|  | **GF9** | Objectives | Also known as missions, side-quests, milestones, etc. This intrinsic concept is related to goals, it provides the player an end, or a purpose to perform the required tasks. Examples on the use of Objective can be broadened (as getting approved in the course) or more specific (as obtaining a certain score in a task). | - First Aid Action Hero^1^ - A Breathtaking Picnic^1^ - ReLIVe Responder^1^ - Responder Rescuebusters: Fire and First-Aid^1^ - Everyday Lifesaver |
|  | **GF10** | Puzzle | Also known as challenges, cognitive tasks, actual puzzles, etc. This intrinsic concept is related to the activities that are implemented within the environment, they can be tied or considered as the learning activities since the focus is to provide a cognitive challenge to the learner. This concept is also implicitly present in all educational environments, through quizzes or challenges. | - First Aid Action Hero - Everyday Lifesaver |
|  | **GF11** | Renovation | Also known as boosts, extra life, renewal, etc. This concept is intrinsically related to the property of re-doing a task, event or any of the sorts. It allows the learner a second chance after they fail a task. It is one of the properties that makes games fun. | - First Aid Action Hero - A Breathtaking Picnic - Responder Rescuebusters: Fire and First-Aid - Everyday Lifesaver^1^ |
|  | **GF12** | Sensation/  Stimulation | This is either visual or sound stimulation, etc. It is related to the use of learners’ senses to improve the experience (intrinsic). This can be done through dynamic and gameful interfaces, virtual reality and/or augmented reality. | - First Aid Action Hero - CPR APP^1^ - A Breathtaking Picnic - ReLIVe Responder - Responder Rescuebusters: Fire and First-Aid - Everyday lifesaver |
|  | **GF13** | Avatar | Also known as player or virtual character. | - First Aid Action Hero - A Breathtaking Picnic - Responder Rescuebusters: Fire and First-Aid - Everyday Lifesaver |
|  | **GF14** | Narrative | Also known as karma system, implicit decisions, etc. This intrinsic concept is the order of events as they happen in the game, through the user experience. This experience is influenced by implicit choices made by the user. Examples of this are giving a small token of appreciation to the students that opt to interact with other students, subtly and discreetly. | - First Aid Action Hero - A Breathtaking Picnic - ReLIVe Responder^1^ - Responder Rescuebusters: Fire and First-Aid - Everyday Lifesaver |
| Performance | **GF15** | Acknowledgement | Also known as badges, medals, trophies, and achievements. It is a kind of extrinsic feedback that praises the players’ specific set of actions, e.g., completing a certain number of problems may lead them to earn a “BLS^b^” badge; finishing a task in a predefined time limit may earn them a “BLS” trophy; making a certain number of interactions with other users may give them a “BLS” achievement; making a certain number of contributions may earn them a “BLS” badge. | - First Aid Action Hero - CPR APP - Responder Rescuebusters: Fire and First-Aid - Everyday Lifesaver |
|  | **GF16** | Level/Stages | Also known as skill level, character level (DRSABCD) etc. This is related to an extrinsic hierarchical layer that provides the user new advantages as they advance in the environment, eg, the user gain a level every time they complete a certain number of tasks, when they advance their level, they have access to more challenging tasks. | - First Aid Action Hero - A Breathtaking Picnic^1^ - ReLIVe Responder - Responder Rescuebusters: Fire and First-Aid - Everyday Lifesaver |
|  | **GF17** | Progression | Also known as progress bars, steps, maps. Provides an extrinsic guidance to the users of their advance in the environment, allowing these users to locate themselves. | - A Breathtaking Picnic^1^ - Responder Rescuebusters: Fire and First-Aid^1^ |
|  | **GF18** | Point(s) | Also known as scores, experience points, skill points, etc. It is a simple way to provide extrinsic feedback to the users’ actions. | - A breathtaking picnic - Responder Rescuebusters: Fire and First-Aid |
|  | **GF19** | Competition | Also known as conflict, leader boards, scoreboards, player vs player, etc. It’s an intrinsic concept, tied to a challenge where the user faces another user to achieve a common goal, eg, using scoreboards based on the number of points, badges, levels, etc. | - First Aid Action Hero^1^ - CPR APP^1^ - A Breathtaking Picnic - ReLIVe Responder - Responder Rescuebusters: Fire and First-Aid^1^ |
|  | **GF20** | Statistics | Also, can be seen as audio queues, text stories, etc. It is the way the story of the environment is told (as a script). It is told through text, voice, or sensorial resources. It is highly used as a tool to support the narrative within an environment | - CPR APP - Responder Rescuebusters: Fire and First-Aid |
|  | **GF21** | History | Also known as shadowing. Giving users the ability to compare their results with previous scores such as correct chest compressions. | - CPR APP^1^ - A Breathtaking Picnic - Responder Rescuebusters: Fire and First-Aid^1^ - Everyday Lifesaver |
|  | **GF22** | Prizes/Rewards | The use of prizes has been found to be effective in motivating learners. | CPR APP^1^ |
| Educational | **GF23** | Storytelling/  Storyline | Also known as information, head up display and data. It is related to the visual information provided by the environment to the learner (extrinsic), eg, how many tasks they completed or overall stats on the environment. In virtual environments this can also be dashboards. | - First Aid Action Hero - CPR APP^1^ - A Breathtaking Picnic - ReLIVe Responder^1^ - Everyday Lifesaver ^1^ |
|  | **GF24** | Feedback | Any kind of visual or audible feedback that is related to BLS contents. | - First Aid Action Hero - CPR APP - A Breathtaking Picnic - ReLIVe Responder - Responder Rescuebusters: Fire & First-Aid - Everyday Lifesaver |
|  | **GF25** | Retention | Also known as choice, judgment, and paths. This extrinsic concept occurs when the player faces an explicit decision that they must make to advance in the environment. An example of this concept is to present the user two different contents and make them choose one or another, blocking their advance if a choice is not to pick. | - First Aid Action Hero - Everyday Lifesaver |
|  | **GF26** | Imposed choice | Any kind of game mechanics (eg, quiz) to maintain user interest of BLS after finishing m-learning. | - First Aid Action Hero - CPR APP^1^ - A Breathtaking Picnic^1^ - ReLIVe Responder^1^ - Responder Rescuebusters: Fire & First-Aid^1^ - Everyday Lifesaver |

^a^ID: Identification number

GF: gamification feature

^b^BLS: Basic life support

^1^ Graded with 0.5 point.
