# Supplementary material for: Evaluating Quality, Usability, Evidence-Based Content, and Gamification Features in Mobile Learning Apps Designed to Teach Children Basic Life Support: Systematic Search in App Stores and Content Analysis
Source: JMIR Mhealth Uhealth. 2021 Jul 20;9(7):e25437. doi: 10.2196/25437 (PMC8335615; doi:10.2196/25437)
Supplement: Multimedia Appendix 4 [file mhealth_v9i7e25437_app4.docx]

**Multimedia Appendix 4:** Description of the included apps.

**Table 1.** Description of the included apps.

| **Full app name** | **Healthcare organization collaborator** | **County, BLS^a^ guideline organization** | **Description of BLS scenarios** |
| --- | --- | --- | --- |
| First Aid Action Hero | St John Ambulance Australia (Victoria) | Australia (St John Ambulance Australia) | One scenario where user performed BLS first on conscious and second on unconscious animated cartoon figure |
| CPR APP | Emergency Medicine Unit, Li Ka Shing Faculty of Medicine, The University of Hong Kong | United States (American Heart Association) | Without scenario. In simulation environment user performed BLS on animated human figure |
| Everyday Lifesaver | Life Saving Victoria Limited | Australia (St. John Ambulance Australia) | Three scenarios where user performed BLS on drowned adult, drowned child and unconscious animated cartoon figure |
| A Breathtaking Picnic | The Italian Resuscitation Council | Italy (The Italian Resuscitation Council) | Two scenarios where user performed BLS on animated animal which is choking and had a cardiac arrest |
| ReLIVe Responder | The University of Pittsburgh, Department of Emergency Medicine | United States (American Heart Association) | Two scenarios where user performed BLS on unconscious and conscious animated human figure |
| Responder Rescuebusters: Fire and First-Aid | Emergency Response Centre Agency Finland, Finnish Recovery Council, Finnish Fire Officers' Association's | Finland (Finnish Recovery Council) | One scenario where user performed BLS on animated human figure which had a cardiac arrest |

^a^BLS: basic life support.
