# Supplementary material for: Evaluating Quality, Usability, Evidence-Based Content, and Gamification Features in Mobile Learning Apps Designed to Teach Children Basic Life Support: Systematic Search in App Stores and Content Analysis
Source: JMIR Mhealth Uhealth. 2021 Jul 20;9(7):e25437. doi: 10.2196/25437 (PMC8335615; doi:10.2196/25437)
Supplement: Multimedia Appendix 5 [file mhealth_v9i7e25437_app5.docx]

**Multimedia Appendix 4:** User version of the Mobile Application Rating Scale scores and sections.

|  | **User version of the Mobile Application Rating Scale scores and sections** | | | | | | | | | | | | | | | | | | |  |
| --- | --- | --- | --- | --- | --- | --- | --- | --- | --- | --- | --- | --- | --- | --- | --- | --- | --- | --- | --- | --- |
|  | **SECTION A:  Engagement** | | | | | **SECTION B: Functionality** | | | | | **SECTION C: Aesthetics** | | | | **SECTION D: Information** | | | | **Overall app mean quality** |  |
| **Full app name** | **Entertainment** | **Interest** | **Customisation** | **Interactivity** | **Target group** | **Performance** | **Ease of use** | **Navigation** | **Gestural design** | **Layout** | | **Graphics** | **Visual appeal** | **Quality of information** | | **Quantity of information** | **Visual information** | **Credibility of source** |  |  |
| **First Aid Action Hero [59]** | 3.9 | 3.9 | 2.8 | 3.8 | 4.0 | 4.3 | 3.9 | 3.9 | 3.9 | 4.3 | | 4.1 | 3.6 | 3.7 | | 3.7 | 3.6 | 4.4 | 3.9 |  |
|  |  |  |  |  |  |  |  |  |  |  |  |  |  |  |  |  |  |  |  |  |
| **CPR APP [60]** | 3.3 | 3.5 | 1.9 | 3.4 | 3.2 | 3.9 | 3.5 | 3.6 | 3.6 | 3.4 | | 3.2 | 2.9 | 4.0 | | 3.4 | 3.4 | 4.1 | 3.4 |  |
|  |  |  |  |  |  |  |  |  |  |  |  |  |  |  |  |  |  |  |  |  |
| **Everyday Lifesaver [61]** | 3.7 | 3.9 | 2.9 | 3.7 | 3.5 | 3.3 | 3.0 | 2.9 | 2.8 | 3.4 | | 3.9 | 3.8 | 3.7 | | 3.7 | 3.5 | 3.1 | 3.4 |  |
|  |  |  |  |  |  |  |  |  |  |  |  |  |  |  |  |  |  |  |  |  |
| **A Breathtaking Picnic [62]** | 3.3 | 3.4 | 2.0 | 2.6 | 3.3 | 3.9 | 3.3 | 3.3 | 3.5 | 3.9 | | 4.0 | 3.9 | 3.5 | | 3.5 | 3.2 | 4.4 | 3.4 |  |
|  |  |  |  |  |  |  |  |  |  |  |  |  |  |  |  |  |  |  |  |  |
| **ReLIVe Responder [63]** | 3.3 | 3.4 | 1.7 | 2.9 | 2.9 | 3.8 | 3.3 | 3.7 | 3.6 | 3.4 | | 3.2 | 2.9 | 3.3 | | 3.1 | 3.6 | 4.1 | 3.3 |  |
|  |  |  |  |  |  |  |  |  |  |  |  |  |  |  |  |  |  |  |  |  |
| **Responder Rescuebusters: Fire and First-Aid [64]** | 3.7 | 3.4 | 2.6 | 3.1 | 3.6 | 3.6 | 3.1 | 3.5 | 3.2 | 3.7 | | 3.7 | 3.5 | 3.1 | | 2.9 | 3.1 | 3.7 | 3.3 |  |
|  |  |  |  |  |  |  |  |  |  |  |  |  |  |  |  |  |  |  |  |  |
