# Supplementary material for: Evaluating Quality, Usability, Evidence-Based Content, and Gamification Features in Mobile Learning Apps Designed to Teach Children Basic Life Support: Systematic Search in App Stores and Content Analysis
Source: JMIR Mhealth Uhealth. 2021 Jul 20;9(7):e25437. doi: 10.2196/25437 (PMC8335615; doi:10.2196/25437)
Supplement: Multimedia Appendix 6 [file mhealth_v9i7e25437_app6.docx]

**Multimedia Appendix 6.** User version of the Mobile Application Rating Scale and time spent on each app results.

**Table 2.** User version of the Mobile Application Rating Scale results and time spent on each app.

| Full app name | uMARS^a^ section | | | | | Time^b^ |
| --- | --- | --- | --- | --- | --- | --- |
|  | Engagement | Functionality | Aesthetics | Information | Overall app quality |  |
|  | | | | | | |
| First Aid Action Hero [59] | 3.7 | 4.0 | 4.0 | 3.87 | 3.9 | 12 |
| CPR APP [60] | 3.1 | 3.7 | 3.2 | 3.7 | 3.4 | 7 |
| Everyday Lifesaver [61] | 3.5 | 3.0 | 3.7 | 3.5 | 3.4 | 24 |
| A Breathtaking Picnic [62] | 2.9 | 3.5 | 4.1 | 3.7 | 3.5 | 5 |
| ReLIVe Responder [63] | 2.8 | 3.6 | 3.2 | 3.5 | 3.3 | 5 |
| Responder Rescuebusters: Fire and First-Aid [64] | 3.4 | 3.4 | 3.6 | 3.2 | 3.4 | 4 |

^a^uMARS: user version of the Mobile Application Rating Scale.

^b^Mean time for testing apps (in min).

**Table 3.** Mean scores and intraclass correlation coefficients of the user version of the Mobile Application Rating Scale and time spent on each app.

| Variable | | Score, mean (95% CI) | ICC_2, k_^a^ (95% CI) |
| --- | --- | --- | --- |
| **uMARS^b^ section** | | | |
|  | Engagement | 3.2 (3.0-3.4) | 0.9 (0.8-0.9) |
|  | Functionality | 3.5 (3.4-3.7) | 0.7 (0.5-08) |
|  | Aesthetics | 3.6 (3.4-3.8) | 0.8 (0.6-0.9) |
|  | Information | 3.6 (3.4-3.8) | 0.8 (0.6-0.9) |
|  | Overall app quality | 3.2 (3.0-3.4) | 0.9 (0.8-0.9) |
| Time for testing apps (min) | | 9.2 (7.7-10.7) | N/A^c^ |

^a^ICC_2,k_: intraclass correlation coefficient; two-way random, average measures, absolute agreement.

^b^uMARS: user version of the Mobile Application Rating Scale.

^c^N/A: not applicable.
